# Neurofilament light chain levels indicate acute axonal damage under bortezomib treatment

**DOI:** 10.1007/s00415-023-11624-2

**Published:** 2023-02-18

**Authors:** Nadine Cebulla, Daniel Schirmer, Eva Runau, Leon Flamm, Sonja Gommersbach, Helena Stengel, Xiang Zhou, Hermann Einsele, Ann-Kristin Reinhold, Bruno Rogalla von Bieberstein, Daniel Zeller, Heike Rittner, K. Martin Kortüm, Claudia Sommer

**Affiliations:** 1 Department of Neurology, University Hospital Würzburg, Würzburg, Germany; 2 Department of Internal Medicine II, University Hospital Würzburg, Würzburg, Germany; 3 Department of Anesthesiology, University Hospital Würzburg, Würzburg, Germany

**Keywords:** Bortezomib, Bortezomib-induced peripheral neuropathy, Neurofilament light chain, Nerve conduction studies, Multiple myeloma, Peripheral neuropathy

## Abstract

**Introduction:**

Bortezomib (BTZ) is a selective and reversible proteasome inhibitor and first line treatment for multiple myeloma (MM). One of the side effects is BTZ-induced peripheral neuropathy (BIPN). Until now there is no biomarker which can predict this side effect and its severity. Neurofilament light chain (NfL) is a neuron specific cytoskeletal protein, of which higher levels can be detected in peripheral blood in case of axon damage. In this study, we aimed to evaluate the relationship between NfL serum levels and characteristics of BIPN.

**Methods:**

We performed a first interim analysis of a monocentric, non-randomized, observational clinical trial including 70 patients (DRKS00025422) diagnosed with MM in the inclusion period of June 2021 until March 2022. Two groups of patients—one with ongoing BTZ treatment at the time of recruiting, and one with BTZ treatment in the past—were compared to controls. NfL in serum was analyzed via the ELLA™ device.

**Results:**

Both patients with previous and ongoing BTZ treatment had higher serum NfL levels than controls, and patients with ongoing BTZ treatment had higher NfL levels than patients with BTZ treatment in the past. Serum NfL levels correlated with electrophysiological measures of axonal damage in the group with ongoing BTZ treatment.

**Conclusion:**

Elevated NfL levels indicate acute axonal damage under BTZ in MM patients.

**Supplementary Information:**

The online version contains supplementary material available at 10.1007/s00415-023-11624-2.

## Introduction

Multiple myeloma (MM) is a plasma cell disorder which accounts for 13% of all hematological malignancies [1]. First line therapy for this disease is bortezomib (BTZ). BTZ is a selective and reversible proteasome inhibitor, which targets the chymotrypsin-like site of the 20S proteolytic core within the 26S proteasome, a fundamental protease for the ubiquitin–proteasome pathway. The ubiquitin–proteasome system plays an important role in the transcriptional regulation of nuclear factor-kB (NF-kB) [2, 3]. In turn, proteasome inhibition leads to apoptosis of the cancer cells without a toxic effect on most non-cancer cells [4]. BTZ is administered either by intravenous (i.v.) or subcutaneous (s.c.) route, whereby s.c. BTZ application is not inferior in efficacy to i.v. application but has an improved safety profile [5]. One of the side effects of BTZ treatment is BTZ-induced peripheral neuropathy (BIPN). BIPN is a length-dependent sensory axonopathy with numbness, tingling, pain and paresthesia in a symmetrical stocking and glove distribution [6]. Patients suffering from BIPN have various sensory deficits (warmth and heat thresholds, touch and sharpness detection) and in some more severe cases, even pareses [7]. Electrophysiologically, BIPN is a predominantly sensory, axonal polyneuropathy with low or absent amplitude of sensory nerve action potentials (SNAP) and, in more severe cases, reduced amplitude of compound muscle action potentials (CMAP) [6]. Histopathological changes, such as alterations of Schwann cells and myelin as well as axon degeneration, have been described in BIPN rat models [8, 9]. Axonal degeneration after BTZ administration was also described in cultured dorsal root ganglion neurons [10].

Symptoms may regress after the end of the treatment, but BIPN is a dose limiting complication of BTZ treatment, and in some cases, treatment needs to be discontinued [11]. Until now there is no biomarker, which can predict this side effect and its severity. A potential biomarker that can be determined easily is neurofilament light chain (NfL). NfL is a neuron specific cytoskeletal protein, which is important for the cell structural stability in neurons of central and peripheral nervous system (CNS and PNS) [12, 13]. If an axon is damaged, higher amounts of NfL can be detected in peripheral blood [14]. NfL has been extensively studied using established detection methods like SIMOA™ and ELLA™ as a biomarker in various CNS-diseases (e.g. dementia, multiple sclerosis, amyotrophic lateral sclerosis) [15]. During chemotherapy with paclitaxel in breast cancer patients, NfL levels were higher in patients with grade 2–3 than in those with grade 0–1 neuropathy [16].

NfL levels have not yet been reported in patients with BTZ treatment. We investigated whether there are differences in NfL levels in the patients with ongoing BTZ treatment compared to those who have completed their BTZ treatment and to healthy controls, and whether NfL levels would correlate to measures of axonal damage and severity of the neuropathy.

## Patients and methods

### Study design and recruitment

Patients were recruited at the Multiple Myeloma Center of the Department of Internal Medicine II at the University Hospital Würzburg. This is the first interim analysis of a monocentric, non-randomized, observational clinical trial including 70 patients (DRKS00025422). All patients were diagnosed with MM according to current International Myeloma Working Group (IMWG) criteria [17]. The inclusion period for this interim analysis was June 2021 until March 2022. Inclusion criteria were minimum age of 18 years, and the confirmed diagnosis of MM. Exclusion criteria were serious other medical conditions that would prevent the subject from study participation.

The patients were divided into 2 groups: 1. Patients with ongoing treatment with BTZ at time of recruitment (OT), 2. Patients with BTZ treatment in the past (PT).

The Ethics Committee of the Medical Faculty of the University of Würzburg approved the study (# 98/20). After oral and written information, discussion, and a reflection period of at least 24 h, patients who agreed to participate signed the consent form and were thus included in the study. Controls for NfL detection in the serum were healthy volunteers without pain or other known neurological conditions (Ethics approval # 242/17).

### Clinical assessment

MM was classified according to the type of pathologically increased monoclonal protein [1]. Data on previous diagnostic and treatment regimens prior to the admission at our center, were extracted from the patients’ records. The approximate cumulative BTZ dose was determined using information on the number of BTZ applications at our center and, if applicable, at other sites. All patients underwent a standardized neurological examination at the Department of Neurology at the University Hospital Würzburg including a detailed sensory examination. These examinations were performed by CS and, after adequate training, by DS, ER, and LF. Rater competence and reproducibility of findings was assessed at monthly intervals by the supervisor (CS). Muscle strength was evaluated according to the Medical Research Council (MRC) scale. The Overall Disability Sum Score (ODSS) [18] and the modified Toronto Clinical Neuropathy Score (mTCNS) were used. To assess the pain qualities we used the German version of the Neuropathic Pain Symptom Inventory (NPSI) [19]. Quantitative sensory testing (QST) was performed according to the standardized protocol of the German Research Network on Neuropathic Pain (Deutscher Forschungsverbund Neuropathischer Schmerz) [20] using a MSA thermal stimulator (Somedic, Hörby, Sweden), and related to a normative dataset from 180 healthy subjects [21]. For all patients, nerve conduction studies (NCS) on the right sural, median and tibial nerves were performed following standard procedures [22]. In two patients the ulnar nerve instead of the median nerve was studied. Antidromic stimulation was used for sensory NCS. CMAPs and the sural SNAP were recorded by surface electrodes, while the median [ulnar] SNAP was obtained by ring electrodes. One patient was excluded from electrophysiological measurement because of neuropathy caused by vascular occlusion on the side of measurement.

### Grading of neuropathy severity

We established a summary report form for our study patients. This report form included 4 parameters (sensory and motor function, QST, electrophysiology) which were each defined as abnormal or normal. The criteria for an abnormal finding are shown in Table 1. A neuropathy was diagnosed if any of the 4 parameters was pathological. Neuropathies were classified into severity grades, according to the Velcade SmPC [23] shown in Table 2.

**Table 1:** Criteria for abnormal finding

Examination	Abnormal finding	
Sensory function	Deficits in ≥ 1 of the following tests: touch detection, pinprick detection, vibration sensation	
Motor function	Paresis in ≥ 1 muscle group	
QST	≥ 2 pathologic thresholds	
Electrophysiology	Pathologic pattern	Criteria
	Probably axonal	1 pathologic nerve (SNAP/CMAP)
	Axonal	≥ 2 pathologic nerves (SNAP/CMAP)
	Demyelinating	Reduced NCV ≥ 2 nerves

**Table 2:** Classification of severity grades

Grade*	Criteria
Severity grade 1	Asymptomatic; loss of deep tendon reflexes or paresthesia
Severity grade 1 with pain	
Severity grade 2	Moderate symptoms; limiting instrumental activities of daily living (ADL)**
Severity grade 2 with pain	
Severity grade 3	Severe symptoms; limiting self-care ADL***
Severity grade 4	Life-threatening consequence; urgent intervention indicated and/or severe autonomic neuropathy

*Based on posology modifications in Phase II and III multiple myeloma studies and post marketing experience. Grading based on NCI Common Toxicity Criteria CTCAE v 4.0

**Instrumental ADL: refers to preparing meals, shopping for groceries or clothes, using telephone, managing money, etc.

***Self-care ADL: refers to bathing, dressing and undressing, self-feeding, using the toilet, taking medications, and not confined to bed

### Measurement of NfL concentration in serum

Venous blood was drawn in the morning and allowed to stand for 30 min. Then it was centrifuged at 1200×*g* for 10 min and the serum was stored at − 20° and thawed immediately before use. NfL levels were measured using the ELLA™ device (ProteinSimple, CA, USA), a next generation Enzyme-Linked Immunosorbent Assay (ELISA), according to manufacturer’s instructions by a blinded investigator. Quantification range was 2.7 − 10,290 pg/ml.

### Statistics

Statistical analyses were performed using IBM SPSS Statistics Version 28.0 (Armonk, New York, USA) and Prism Version 9.4.1 (GraphPad Software, San Diego, CA). Graphs were created with Prism. Normality of the data was tested by Shapiro-Wilk normality test. Statistical significance was calculated using either an unpaired two-tailed Mann-Whitney test for pairwise comparison or an unpaired Kruskal-Wallis-Test with Dunn’s correction for group comparisons, depending on the data sets to be analyzed. The correlation was determined with the Spearman correlation coefficient. Statistical significance was set at *p* < 0.05.

## Results

### Demographics

Our interim analysis cohort consisted of 70 patients (50 men); the median age was 66 years (range 31–82). Table 3 shows all demographic data. Sixty-three patients were affected by neuropathy (30 in the OT, 33 in the PT group). Most patients had mild neuropathy (Table 3), but 33/63 were painful. We included 18 healthy controls (10 men), with a median age of 61 years (range 21–73).

**Table 3:** Demographic, clinical data of the cohort

Number of patients		Overall cohort	Ongoing BTZ treatment (OT)	BTZ treatment in the past (PT)
		70	34	36
Sex	Male/female	50/20	24/10	26/10
Age in years	Median	66	64	66
	*(Range)*	*(31–82)*	*(47–81)*	*(31–82)*
Neuropathy		63	30	33
Painful neuropathy		33	12	21
Severity grades				
Grade 1		22	12	10
Grade 1 with pain		14	6	8
Grade 2		7	5	2
Grade 2 with pain		17	6	11
Grade 3 (with pain)		3 (2)	1	2 (2)
Abnormal findings				
Sensory function		61	29	32
Motor function		25	9	16
QST^a^		52	24	28
Electrophysiology				
Probably axonal		14	9	5
Axonal		33	11	22
Demyelinating		2	0	2
Myeloma classification				
Heavy chain IgA		12	6	6
Heavy chain IgG		39	19	20
Heavy chain IgM		1	1	0
Light chain kappa LC		40	21	19
Light chain lambda LC		26	14	12
ODSS^b^	Median	2	1.5	2
	*(Range)*	*(0–7)*	*(0–7)*	*(0–6)*
MRC^c^ sum score	Median	120	120	120
	*(Range)*	*(99–120)*	*(102–120)*	*(99–120)*
mTCNS^d^ sum score	Median	11	10.5	13
	*(Range)*	*(0–29)*	*(0–23)*	*(0–29)*
NPSI^e^ sum score	Median	10	8	18
	*(Range)*	*(0–91)*	*(0–51)*	*(0–91)*
Disease duration in months	Median	44.0	34.5	44
	*(Range)*	*(2–205)*	*(2–163)*	*(4–205)*
Cumulative dose (mg/m^2^)	Median	22.8	33.4	20.8
	*(Range)*	*(3.9–227.2)*	*(8.6–153.4)*	*(3.9–227.2)*
Time since last application in months	Median	2	0	16.5
	*(Range)*	*(0–115)*	*(0–6)*	*(1–115)*

Italic values displays the range

^a^*QST* Quantitative sensory testing

^b^*ODSS* Overall disability sum score

^c^*MRC* Medical research council

^d^*mTCNS* Modified toronto clinical neuropathy score

^e^*NPSI* Neuropathic pain symptom inventory

### NfL levels

**Fig. 1:**
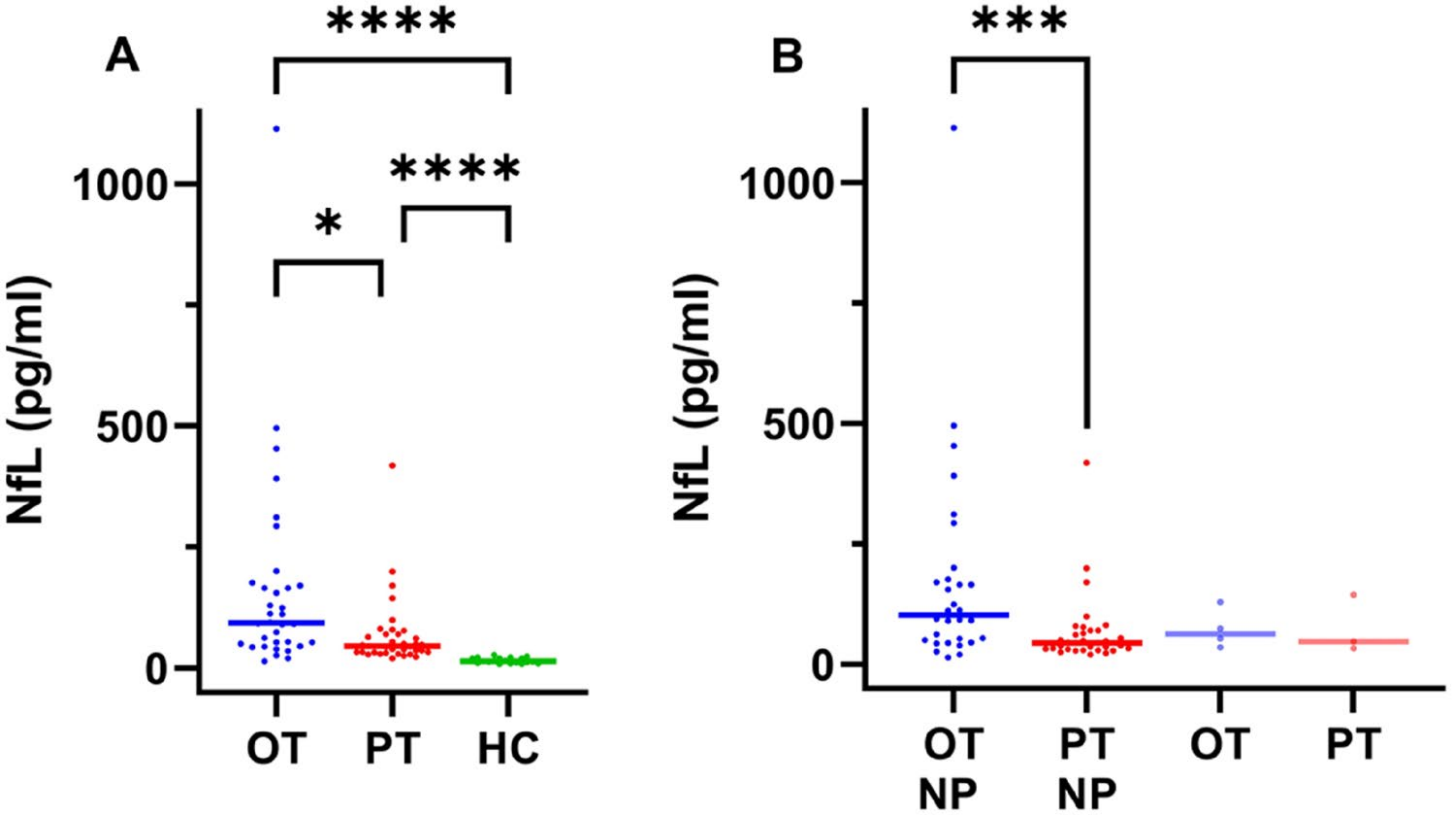
**A** Median NfL levels in the cohorts with ongoing BTZ treatment (OT) and the BTZ treatment in the past (PT) and healthy controls (HC); (OT) *N* = 34, 93.4 pg/ml, (PT) *N* = 36, 45.3 pg/ml, (HC) *N* = 18, 14.8 pg/ml; group comparisons were performed with Kruskal–Wallis-Test and Dunn’s correction, **B** Median NfL levels in patients with neuropathy (NP) in the OT NP and in the PT NP subgroup and in the OT and PT subgroup without NP; OT NP (*N* = 30; 102.6 pg/ml), PT NP (*N* = 33; 44.8 pg/ml), OT (*N* = 4; 63.7 pg/ml), PT (*N* = 3; 47.1 pg/ml); Mann–Whitney test was used for pairwise comparison; significance levels: * < 0.05, *** < 0.001, **** < 0.0001

Median NfL levels were higher in both the OT and the PT patient group (OT: 93.4 pg/ml; PT: 45.3 pg/ml) than in the controls (14.8 pg/ml) (*p* =  < 0.0001). Median NfL levels were higher in OT compared to PT group (*p* = 0.03), see Table 4 for detailed data and Fig. 1A for group comparisons. In the OT group, NfL levels were related to the severity grade of the neuropathy. In the PT group, NfL levels were not related to the severity grade, however, one patient had grade 3 and very high NfL level of 418.0 pg/ml. Median NfL levels in patients with neuropathy were 102.6 pg/ml in the OT and 44.8 pg/ml in the PT group (*p* =  < 0.001). The median NfL levels in the patients without neuropathy were 63.7 pg/ml in the OT and 47.1 pg/ml in the PT group (Fig. 1B).

**Table 4:** NfL levels in controls and myeloma patients

		Myeloma patients		
	Healthy controls (*N* = 18)	Overall cohort (*N* = 70)	Neuropathy cohort (*N* = 63)	No neuropathy (*N* = 7)
NfL median (pg/ml)	**14.8**	**54.4**	**54.5**	**53.3**
*(Range)*	*(8.1–27.8)*	*(14.4–1114.0)*	*(14.5–1114.0)*	*(33.8–144.0)*

Ongoing treatment (OT)		BTZ treatment in the past (PT)		*p*
Overall	**93.4**	Overall	**45.3**	**0.0007**^a^
*N* = 34	*(14.5–1114.0)*	*N* = 36	*(20.0–418.0)*	
No neuropathy	**63.7**	No neuropathy	**47.1**	**0.8571**^a^
*N* = 4	*(35.5–129.0)*	*N* = 3	*(20.0–418.0)*	
Grade 1	**53.8**	Grade 1	**42.8**	**0.3136**^a^
*N* = 12	*(14.5–495.0)*	*N* = 11	*(23.8–199.0)*	
Grade 1 with pain	**124.6**	Grade 1 with pain	**47.4**	**0.0047**^a^
*N* = 6	*(53.2–1114.0)*	*N* = 7	*(20.0–79.3)*	
Grade 2	**91.6**	Grade 2	**43.5**	**–**
*N* = 5	*(50.7–391.0)*	*N* = 2	*(38.4–48.7)*	
Grade 2 with pain	**144.5**	Grade 2 with pain	**44.3**	**0.0616**^a^
*N* = 6	*(20.6–311.0)*	*N* = 11	*(29.6–170.0)*	
Grade 3	**111**	Grade 3	**223.1**	**–**
*N* = 1		*N* = 2	*(28.2–418.0)*	

Italic values display the range. Bold values displays the Median NfL levels in this table. In the right column bold displays the *p* value

^**a**^Mann-Whitney test

### Axonal damage and NfL levels

To assess whether increased NfL levels are related to measures of axonal damage, we divided the patients into subgroups according to NCS data. Table 5 displays the NCS data of the overall cohort and the subgroups. In the PT group sural nerve SNAPs and median nerve SNAPs and CMAPs had lower amplitudes than in the OT group.

**Table 5:** Nerve conduction studies of the overall cohorts and the subgroups, which were separated according to classification (Table 1) of measurements to normal or abnormal

	Ongoing BTZ treatment (OT)						BTZ treatment in the past (PT)					
	Overall	*N*	Normal	*N*	Axonal	*N*	Overall	*N*	Normal	*N*	Axonal	*N*
Sural nerve												
SNAP^a^ µV (median)	5.9	33	10.9	13	1.4	11	2.8	36	11.5	7	0.9	22
NCV^b^ m/s (median)	44.8	33	48.1	13	41.0	11	43.6	36	48.1	7	19.3	22
Tibial nerve												
CMAP dis^c^ mV (median)	11.6	33	16.4	13	5.3	11	8.0	36	15.9	7	4.9	22
NCV m/s^b^ (median)	44.3	33	45.9	13	44.1	11	41.4	36	46.1	7	40.7	22
Median Nerve												
CMAP dis^c^ mV (median)	11.5	32	13.0	12	9.9	11	10.6	35	11.3	7	9.8	22
NCV^b^ m/s (median)	53.6	32	54.6	12	52.9	11	53.7	35	56.1	7	53.3	22
SNAP^a^ µV (median)	17.6	32	25.1	12	14.0	11	13.3	35	30.4	7	9.9	22
NCV^b^ m/s (median)	53.9	32	56.8	12	51.3	11	54.4	35	51.9	7	47.6	22

^a^*SNA*P Sensory nerve action potential

^b^*NCV* Nerve conduction velocity

^c^*CMAP* dis compound muscle action potential after distal stimulation

**Fig. 2:**
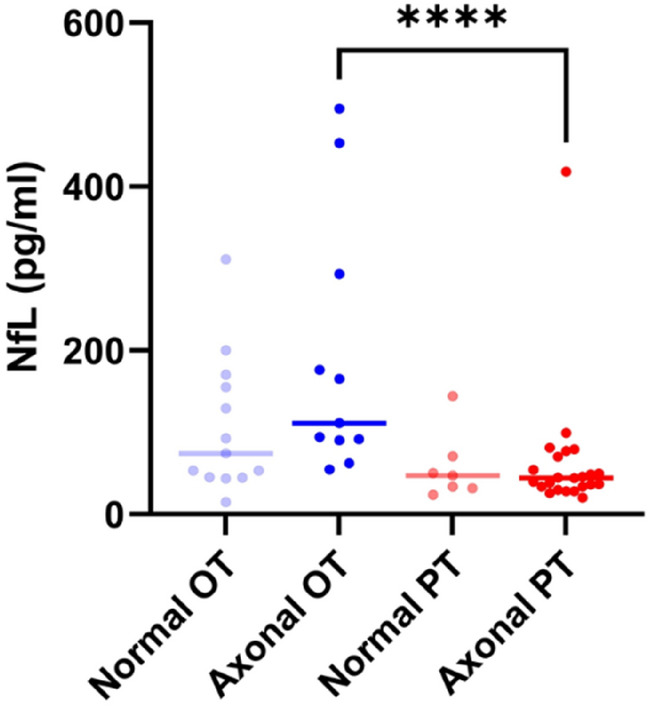
Median NfL levels of subgroup with an axonal damage pattern and the subgroup with normal NCS findings; OT: ongoing BTZ treatment; PT: BTZ treatment in the past; normal: no pathological finding; axonal: ≥ 2 pathologic nerves (SNAP/CMAP); Normal OT (*N* = 13; 74.1 pg/ml), Axonal OT (*N* = 11; 111.0 pg/ml), Normal PT (*N* = 7; 47.1 pg/ml), Axonal PT (*N* = 23; 44.3 pg/ml); Mann–Whitney test was used for pairwise comparison; significance levels: **** < 0.0001

In the OT group median NfL levels were slightly higher in the subgroup with an axonal damage pattern (111.0 pg/ml) compared to the subgroup with normal NCS findings (74.1 pg/ml), but the difference was not significant (*p* = 0.09). In the PT group there was no difference between these subgroups. However, median NfL levels were higher in the OT subgroup with axonal damage (111.0 pg/ml) compared to the PT subgroup with axonal damage (44.3 pg/ml) (*p* =  < 0.0001) (Fig. 2).

**Fig. 3:**
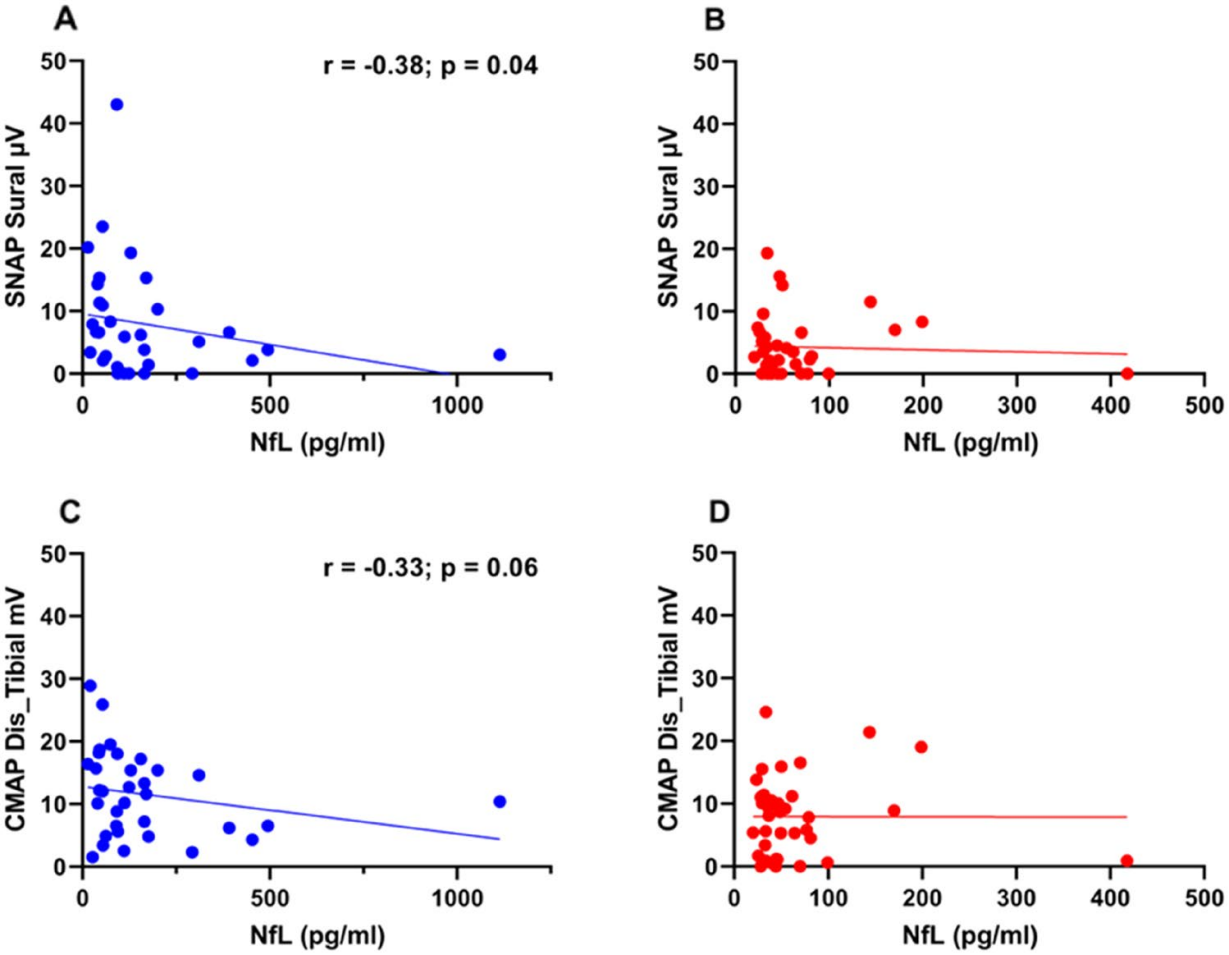
**A** Correlation of the NfL levels and sensory nerve action potential (SNAP) of the sural nerve OT group (Spearman correlation: *r* = − 0.38; *p* = 0.04; *N* = 33), **B** Correlation of the NfL levels and sensory nerve action potential (SNAP) of the sural nerve PT group, **C** Correlation of the NfL levels and compound muscle action potential (CMAP) after distal nerve stimulation of the tibial nerve OT group (Spearman correlation: *r* = − 0.33; *p* = 0.06; *N* = 33), **D** Correlation of the NfL levels and cumulative muscle action potential (CMAP) after distal nerve stimulation of the tibial nerve PT group

When correlating the peak-to-peak amplitude of the sural nerve SNAP with NfL levels, we found a negative correlation of the SNAP amplitude (*r* = − 0.38; *p* = 0.04) with NfL levels in the OT group (Fig. 3A), but not in the PT group (Fig. 3B).

Next, we correlated the amplitude of the tibial nerve CMAP with the NfL levels. We found a trend towards a negative correlation of CMAP (*r* = − 0.33; *p* = 0.06) with NfL levels in the OT group (Fig. 3C). This trend was not found in the PT group (Fig. 3D).

### NfL and cumulative dose or time since last application

**Fig. 4:**
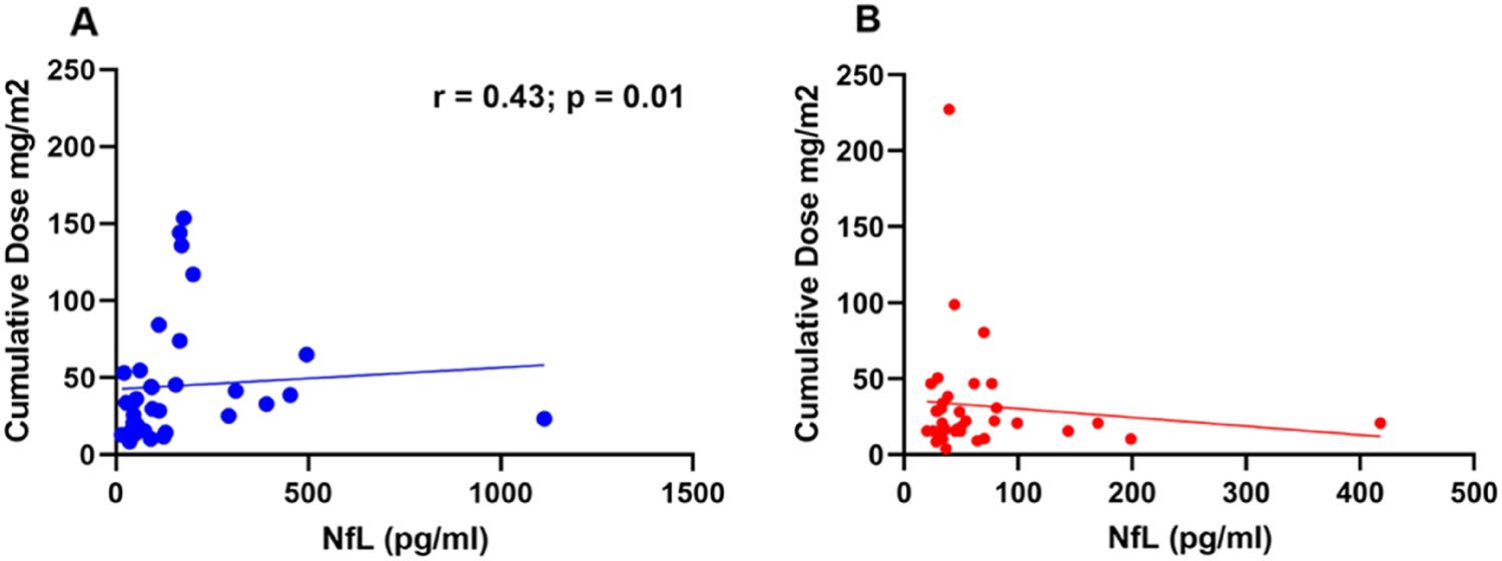
**A** Correlation of cumulative dose and NfL levels in the OT group; (Spearman correlation: *r* = 0.43; *p* = 0.01; *N* = 34), **B** Correlation of cumulative dose and NfL levels in the PT group

**Fig. 5:**
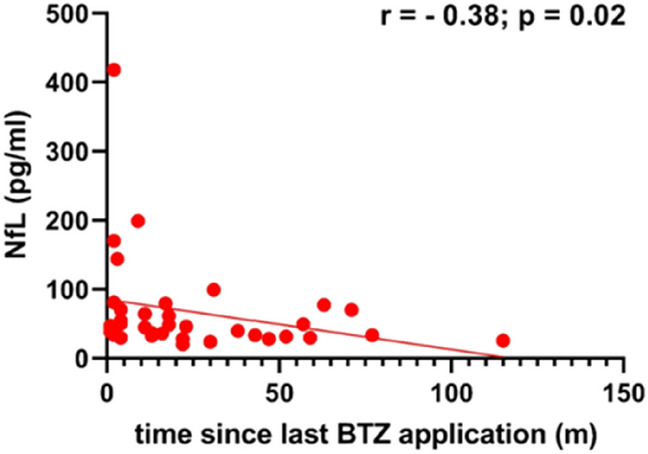
Correlation of the time since the last BTZ application in months and NfL levels in the PT group; (Spearman correlation: *r* = − 0.38; *p* = 0.02; *N* = 36)

To answer the question whether the BTZ cumulative dose is related to NfL levels, we correlated the NfL levels with the approximate cumulative dose. We found a correlation in the OT group (*r* = 0.43, *p* = 0.01) (Fig. 4A), but not in the PT group (Fig. 4B). To test whether the NfL level is related to the time interval since the last BTZ application, we correlated the interval from the last BTZ application until blood collection (in months) with the NfL levels of the PT group. There was a negative correlation (r = − 0.38; *p* = 0.02) between the interval and the NfL levels (Fig. 5).

### NfL and pain, NfL and age

**Fig. 6:**
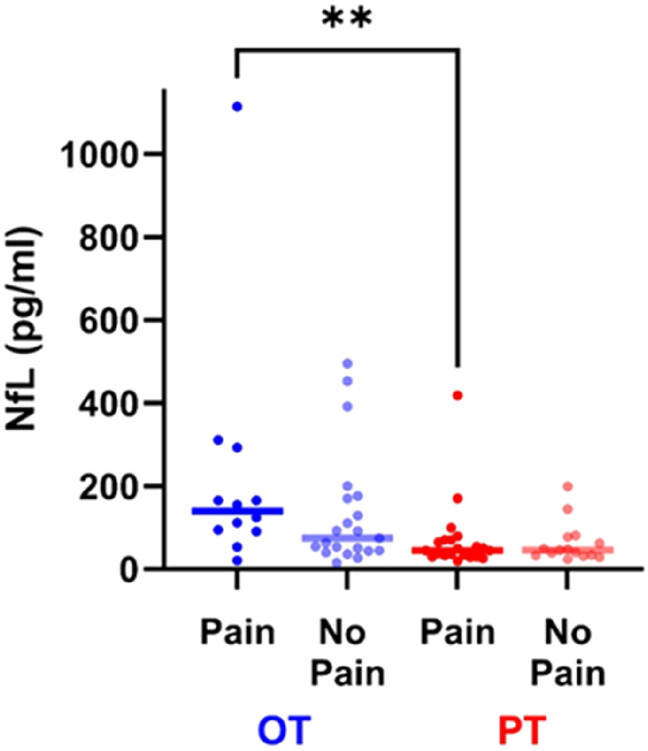
Median NfL levels of subgroup with pain and the subgroup without pain OT: ongoing BTZ treatment; PT: BTZ treatment in the past; Pain OT (*N* = 12; 139.5 pg/ml), No Pain OT (*N* = 21; 74.1 pg/ml), Pain PT (*N* = 21; 44.8 pg/ml), No Pain PT (*N* = 15; 45.9 pg/ml); Mann–Whitney test was used for pairwise comparison; significance levels: ** < 0.01

**Fig. 7:**
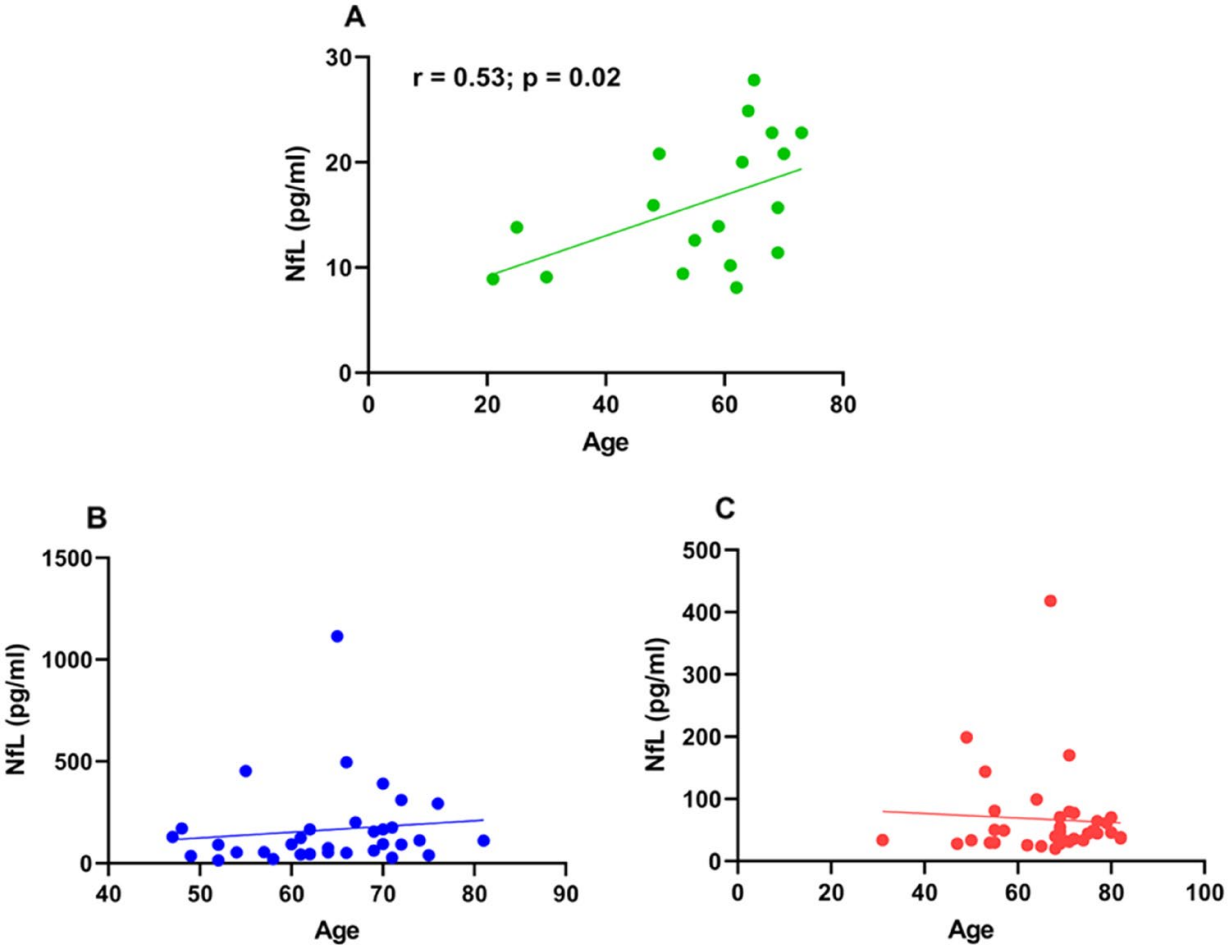
**A** Correlation of the NfL levels and age in years in healthy controls; (Spearman correlation: *r* = 0.53; *p* = 0.02; *N* = 18), **B** Correlation of the NfL levels and age in years OT group, **C** Correlation of the NfL levels and age in years in the PT group

In the OT group, the increase in the subgroup with pain (median NfL level 139.5 pg/ml) compared to the subgroup without pain (74.1 pg/ml) was not significant (*p* = 0.18). In the PT group the subgroup with pain (44.8 pg/ml) was not different from the subgroup without pain (45.9 pg/ml) (*p* = 0.95). Median NfL was higher in the OT subgroup with pain than in the PT subgroup with pain (*p* = 0.002) (Fig. 6). NfL correlated with age in the HC group (*r* = 0.53; *p* = 0.02) (Fig. 7A), as expected, but not in the patient groups (Fig. 7B, C).

### Illustrative case reports

When the individual NfL levels of the two groups were considered, one patient in each group stood out with particularly high NfL levels, which were explored in more detail.

In the OT group, there was a 65 year old male patient with an NfL level of 1114.0 pg/ml and severity grade 1 with pain, see data point at 1114.0 pg/ml in Fig. 4. This patient was diagnosed in July 2021 and until the time of inclusion into the study in November 2021, he had received 4 cycles of BTZ therapy, an approximate cumulative dose of 23.4 mg/m^2^. The patient had a history of hypertension and prostate carcinoma, which was completely resected by prostatectomy. The patient reported slight tingling in the soles of the feet after the 2nd cycle, and after the 3rd cycle he had neuropathic pain in the soles of the feet and intermittent loss of sensation in the fingertips. At the day of examination, he rated average pain as 2 and maximum pain as 9 on a numeric rating scale (NRS) from 0 to 10. He had stocking-like cold and heat hypoesthesia and a mTCNS score of 15, but did not report any impairments in daily life at this time (ODSS = 0). SNAP amplitude of sural nerve was 3.0 µV, CMAP amplitude after distal stimulation of the tibial nerve was still normal. What was striking here, was the relatively early onset of symptoms after only 2 cycles of therapy, associated with a very high NfL level.

A 67 year old male patient in the PT group had a severity grade of 3 and an NfL level of 418.0 pg/ml. This patient was diagnosed with MM in June 2021, had 4 cycles of therapy and an approximate cumulative dose of 20.8 mg/m^2^. The patient had a history of atrial fibrillation. He was severely affected by the neuropathy at the time of examination in December 21. He reported sudden onset of severe pain and motor deficits after the 3rd BTZ cycle. He suffered from neuropathic pain average of 2, maximum 7 on the NRS, he had hypoesthesia and hypalgesia of the feet and stocking-like cold and heat hypoesthesia. He had an MRC score of 99, a mTCNS score of 29 and an ODSS score of 6. The sural nerve showed no response, and CMAP amplitude after distal stimulation of the tibial nerve was 0.9 mV. Even the SNAP of the median nerve was affected (7.8 µV). BTZ therapy was stopped 2 months before inclusion. Considering this fact, we can assume that the NfL level would have been much higher at that time point.

## Discussion

In this cross-sectional analysis, we found that patients with BTZ treatment either at present or in the past had higher serum NfL levels than controls, and that patients with ongoing BTZ treatment had higher NfL levels than patients with BTZ treatment in the past. There was a trend towards an increase in NfL levels across neuropathy severity grades in the OT group, and NfL levels correlated with neurophysiological measures of axonal damage.

In this analysis, we included patients with BTZ therapy with and without neuropathy, to first analyze the effect of BTZ on NfL levels in general. In our cohort, more patients had neuropathy than described in the literature; there were only 4 patients in the OT and 3 in the PT group without neuropathy. However, the OT patients were included into the study within the first 5 BTZ cycles. Whether these patients will develop neuropathy later will be the question of a follow-up study, providing longitudinal data, and thus also information on the relation of NfL levels and the course of BIPN.

Our clinical findings are in agreement with the disease pattern of BIPN described before [6] and the known effects of BTZ in animal models [8, 9]. We confirmed that BIPN is a predominantly sensory, axonal polyneuropathy with low or absent sensory action potentials and, in more severe cases, reduced CMAP amplitudes [6]. We could clearly show that BTZ induced acute axonal damage in patients with current BTZ treatment, indicated by both neurophysiological findings and NfL levels. Although the PT group had even lower SNAP and CMAP amplitude values in the sural and tibial nerves, on average, they had lower NfL levels than the OT group. This might be explained by BTZ-triggered acute axonal damage that leads to NfL release into the blood early in the course of BIPN. At later stages, even if axonal damage persists, this is no longer reflected by the NfL levels. Thus, the elevated NfL levels clearly indicate the acute stage of axonal damage under BTZ treatment in multiple myeloma patients.

Neurotoxicity in general is often related to the cumulative dose of chemotherapeutic agents [24]. Here, we found increased NfL levels with increasing cumulative doses in the OT group, but not in the PT group. Thus, acute axon damage is dose dependent, but this effect is not seen in patients who have completed treatment. Follow-up studies will be needed to find out if there is a long-term dose-dependence in BIPN. By correlating the time since the last application and the NfL levels, we could show that NfL levels decrease with time. Our data are consistent with those from a study of oxaliplatin-treated patients, where the NfL levels decreased 4–6 months after completion of chemotherapy, whereas SNAP did not recover [25].

NfL has recently been investigated in patients receiving other neurotoxic agents like vincristine and paclitaxel [13, 16, 25–29]. So far, all studies concur in the finding of a relation of NfL levels to axonal damage, and in the few longitudinal studies, even a predictive value has been seen [25–27]. From these findings it appears that NfL can indicate axonal damage in chemotherapy irrespective of the specific neurotoxic mechanism of the drug.

Our study has some limitations, first, its cross-sectional design. A longitudinal evaluation will be needed to assess the predictive value of NfL for BIPN. Furthermore, patients received individual therapy regimens in combination with BTZ based on myeloma parameters and the patient's constitution (Suppl. Table 1).

The question also arises whether different pathophysiological processes take place in the acute and chronic phases of BIPN: although the PT group was somewhat more severely affected, the patients from this group nevertheless had NfL values that are closer to the control values, the longer the time since the last application. Follow up of patients in whom BIPN does not regress even months to years after discontinuation of BTZ is warranted.

In conclusion**,** this interim analysis established NfL as an indicator of acute axonal damage under BTZ treatment. Measuring NfL in patients receiving BTZ treatment might be used as an early warning sign that dose reduction may be warranted.

## Supplementary Information

Below is the link to the electronic supplementary material.

Supplementary file1 (DOCX 15 KB)

## Data Availability

The datasets generated during and/or analysed during the current study are available from the corresponding author on reasonable request.
