# Supplementary material for: Neurofilament light chain levels indicate acute axonal damage under bortezomib treatment
Source: J Neurol. 2023 Feb 18;270(6):2997–3007. doi: 10.1007/s00415-023-11624-2 (PMC10188420; doi:10.1007/s00415-023-11624-2)
Supplement: Supplementary file 1 — Supplementary file1 (DOCX 15 KB) [file 415_2023_11624_MOESM1_ESM.docx]

**Supplementary Information (SI)**

Neurofilament light chain levels indicate acute axonal damage under bortezomib treatment

Journal of Neurology

Nadine Cebulla, Daniel Schirmer, Eva Runau, Leon Flamm, Sonja Gommersbach, Helena Stengel, Xiang Zhou, Hermann Einsele, Ann-Kristin Reinhold, Bruno Rogalla von Bieberstein, Daniel Zeller, Heike Rittner, K. Martin Kortüm, Claudia Sommer

Department of Neurology, University Hospital Würzburg

Cebulla_N@ukw.de

Suppl. Table 1: Additional chemotherapeutic drugs taken by the individual patients

| **Patient** | Additional drugs | **Patient** | Additional drugs |
| --- | --- | --- | --- |
| **1** | Daratumumab, Doxorubicin, Dexamethasone, Pomalidomide | **39** | Carfilzomib, Cyclophosphamide, Dexamethasone |
| **2** | Daratumumab, Doxorubicin, Dexamethasone, Pomalidomide | **40** | Daratumumab, Cyclophosphamide, Dexamethasone, Lenalidomide |
| **3** | Cyclophosphamide, Doxorubicin, Etoposide, Cisplatin, Dexamethasone, Thalidomide | **41** | Etoposide, Cyclophosphamide |
| **4** | Carfilzomib, Cyclophosphamide, Dexamethasone | **42** | Melphalan |
| **5** | Dexamethasone, Balantamab Mafodotin, Thalidomide | **43** | Daratumumab, Doxorubicin, Dexamethasone |
| **6** | Daratumumab, Carfilzomib, Cisplatin, Etoposide, Cyclophosphamide, Dexamethasone, Thalidomide | **44** | Doxorubicin, Dexamethasone, Pomalidomide |
| **7** | Daratumumab, Doxorubicin, Dexamethasone, Pomalidomide | **45** | Melphalan |
| **8** | Daratumumab, Doxorubicin, Dexamethasone, Lenalidomide | **46** | Daratumumab, Doxorubicin, Dexamethasone, Lenalidomide |
| **9** | Etoposide, Cyclophosphamide | **47** | Carfilzomib, Daratumumab, Dexamethasone |
| **10** | Daratumumab, Doxorubicin, Dexamethasone, Pomalidomide | **48** | Therapy break |
| **11** | No therapy | **49** | Melphalan |
| **12** | Carfilzomib, Cyclophosphamide | **50** | Daratumumab, Doxorubicin, Dexamethasone, Pomalidomide |
| **13** | Etoposide, Cyclophosphamide | **51** | Daratumumab, Dexamethasone, Thalidomide |
| **14** | Daratumumab, Doxorubicin, Dexamethasone, Pomalidomide | **52** | Daratumumab |
| **15** | Daratumumab, Doxorubicin, Dexamethasone, Pomalidomide | **53** | Carfilzomib, Daratumumab, Cyclophosphamide, Dexamethasone |
| **16** | Daratumumab, Doxorubicin, Dexamethasone, Lenalidomide | **54** | None |
| **17** | Daratumumab, Doxorubicin, Dexamethasone, Pomalidomide | **55** | None |
| **18** | Carfilzomib, Daratumumab, Dexamethasone, Lenalidomide | **56** | Lenalidomide |
| **19** | Daratumumab, Carfilzomib, Cisplatin, Etoposide, Cyclophosphamide, Dexamethasone, Thalidomide | **57** | Daratumumab, Doxorubicin, Dexamethasone, Lenalidomide |
| **20** | Melphalan | **58** | Dexamethasone |
| **21** | Daratumumab, Lenalidomide, Dexamethasone | **59** | Daratumumab, Dexamethasone, Thalidomide |
| **22** | Cyclophosphamide, Doxorubicin, Etoposide, Cisplatin, Dexamethasone, Thalidomide | **60** | Daratumumab, Dexamethasone, Thalidomide |
| **23** | Melphalan | **61** | No therapy |
| **24** | Daratumumab, Doxorubicin, Dexamethasone, Pomalidomide | **62** | Daratumumab, Dexamethasone, Thalidomide |
| **25** | Daratumumab, Doxorubicin, Dexamethasone, Lenalidomide | **63** | Lenalidomide |
| **26** | Daratumumab, Doxorubicin, Dexamethasone, Pomalidomide, Pembrolizumab | **64** | None |
| **27** | No therapy | **65** | Lenalidomide |
| **28** | Daratumumab, Doxorubicin, Dexamethasone, Lenalidomide | **66** | Daratumumab, Dexamethasone, Lenalidomide |
| **29** | Lenalidomide | **67** | Lenalidomide |
| **30** | Cyclophosphamide | **68** | None |
| **31** | Dexamethasone, Balantamab Mafodotin | **69** | Ixazomib, Lenalidomide, Dexamethasone |
| **32** | Daratumumab, Dexamethasone, Lenalidomide | **70** | None |
| **33** | None |  |  |
| **34** | None |  |  |
| **35** | Lenalidomide |  |  |
| **36** | Daratumumab, Doxorubicin, Dexamethasone, Pomalidomide |  |  |
| **37** | Ifosfamide, Uromitexane, Etoposide, Epirubicine |  |  |
| **38** | Daratumumab, Doxorubicin, Dexamethasone, Pomalidomide |  |  |
